# Cerebral tissue oxygenation response to brain irradiation measured during clinical radiotherapy

**DOI:** 10.1117/1.JBO.28.1.015002

**Published:** 2023-01-31

**Authors:** Teemu Myllylä, Vesa Korhonen, Priya Karthikeyan, Ulriika Honka, Jesse Lohela, Kalle Inget, Hany Ferdinando, Sakari S. Karhula, Juha Nikkinen

**Affiliations:** a University of Oulu, Research Unit of Health Sciences and Technology, Oulu, Finland; b University of Oulu, Optoelectronics and Measurement Techniques Unit, Oulu, Finland; c Oulu University Hospital, Department of Diagnostic Radiology, Oulu, Finland; d Medical Research Center, Oulu, Finland; e Oulu University Hospital, Department of Oncology and Radiotherapy, Oulu, Finland

**Keywords:** brain, tissue oxygenation, oncology, radiotherapy, functional near-infrared spectroscopy

## Abstract

**Significance:**

Cancer therapy treatments produce extensive changes in the physiological and morphological properties of tissues, which are also individual dependent. Currently, a key challenge involves developing more tailored cancer therapy, and consequently, individual biological response measurement during therapy, such as tumor hypoxia, is of high interest. This is the first time human cerebral haemodynamics and cerebral tissue oxygenation index (TOI) changes were measured during the irradiation in clinical radiotherapy and functional near-infrared spectroscopy (fNIRS) technique was demonstrated as a feasible technique for clinical use in radiotherapy, based on 34 online patient measurements.

**Aim:**

Our aim is to develop predictive biomarkers and noninvasive real-time methods to establish the effect of radiotherapy during treatment as well as to optimize radiotherapy dose planning for individual patients. In particular, fNIRS-based technique could offer an effective and clinically feasible online technique for continuous monitoring of brain tissue hypoxia and responses to chemo- and radiotherapy, which involves modulating tumor oxygenation to increase or decrease tumor hypoxia. We aim to show that fNIRS is feasible for repeatability measuring in patient radiotherapy, the temporal alterations of tissue oxygenation induced by radiation.

**Approach:**

Fiber optics setup using multiwavelength fNIRS was built and combined with a medical linear accelerator to measure cerebral tissue oxygenation changes during the whole-brain radiotherapy treatment, where the radiation dose is given in whole brain area only preventing dosage to eyes. Correlation of temporal alterations in cerebral haemodynamics and TOI response to brain irradiation was quantified.

**Results:**

Online fNIRS patient measurement of cerebral haemodynamics during clinical brain radiotherapy is feasible in clinical environment, and results based on 34 patient measurements show strong temporal alterations in cerebral haemodynamics and decrease in TOI during brain irradiation and confirmed the repeatability. Our proof-of-concept study shows evidently that irradiation causes characteristic immediate changes in brain tissue oxygenation.

**Conclusions:**

In particular, TOI seems to be a sensitive parameter to observe the tissue effects of radiotherapy. Monitoring the real-time interactions between the subjected radiation dose and corresponding haemodynamic effects may provide important tool for the researchers and clinicians in the field of radiotherapy. Eventually, presented fNIRS technique could be used for improving dose planning and safety control for individual patients.

## 1 Introduction

Radiotherapy is a cornerstone of clinical management of various malignancies, such as brain tumours, certain gastrointestinal malignancies, prostate cancer, and head and neck carcinomas. Typically, radiotherapy is delivered using a linear accelerator (LINAC) producing x-ray photons or electron beams. Radiotherapy is often administered with a curative intention, aiming to administer as high dose as possible to tumor tissue while sparing surrounding healthy tissue. Radiotherapy can be delivered by multiple techniques at variable radiation energy levels. Selection of technique and energy is based on tumor properties.

Radiation doses and dose planning for different malignancies founded on historical evidence, clinical studies, and radiobiology. At present, the clinical efficacy of a treatment can only be determined on clinical or radiological responses observed after a significant period of time following radiotherapy. One significant consideration in dose calculation involves potential toxic effects on surrounding healthy tissue. Radiation treatment on brain tumours, in particular, may subacutely or chronically affect cognition and cause fatigue even at conventional doses. In the context of head and neck tumours and gastrointestinal carcinomas (mainly rectal cancer), mucositis is the most frequent dose-limiting factor. These acute and subacute adverse events often lead to severe and highly symptomatic long-term effects and impaired quality of life. There are currently no biological markers available to predict the development or later improvement of these symptoms. The only currently available method of tracing the number of dose errors is *in vivo* dosimetry, which is used to some extend in external beam radiotherapy to detect major dose errors, to assess clinically relevant differences between planned and delivered doses, and to record doses received by individual patients. However, for a variety of reasons, it is not widely applied to verifying dose delivery to individual patients.^1^^,^^2^ Recently, researchers have invested considerable effort into improving image-guided radiotherapies, where computed tomography (CT) allows precise target localization along with tumor and normal structure position verification in three dimensions.^3^^,^^4^ However, finding new approaches to real-time monitoring of tissue responses to radiotherapy treatments, such as changes in tissue oxygenation is of utmost importance.

In recent years, tumor hypoxia has been studied particularly by using positron emission tomography.^5^ Only few studies have been conducted using optics-based methods to investigate tissue response to radiotherapy, particularly hemodynamic and blood flow related parameters, reviewed by Yu.^6^ A pilot study in 2006 by Sunar et al. measured tumor blood flow and oxygenation response in head and neck chemoradiotherapy using diffuse correlation spectroscopy (DCS) and functional near-infrared spectroscopy (fNIRS). The measurements were conducted before radiotherapy and after each weekly treatment. In the first two weeks, after the treatment considerable changes in relative blood flow, tissue oxygen saturation (${\mathrm{StO}}_{2}$) and total hemoglobin (HbT) were observed.^7^ Similarly, Dong et al. used NIRS/DCS setup and studied more head and neck tumor blood flow and hemodynamic effects of chemoradiotherapy in a larger number of patients (n=47), and likewise, distinctive changes in tumor hemodynamics were found within the first 3 weeks of treatment.^1^ The immediate hemodynamic effects of radiotherapy, however, have yet not been studied, which is probably due to the lack of appropriate clinically approved technologies.

In this paper, we study immediate cerebral haemodynamic and tissue oxygenation changes during clinical brain radiotherapy. At present, more than 50 patients online fNIRS measurements have been performed in Oulu University Hospital, Finland, indicating that the NIRS technique is feasible for clinical patient monitoring in radiotherapy. Our first initial study was presented in 2020 by Myllylä et al.^8^ This paper studied further characteristic temporal alterations in cerebral haemodynamics based on 34 patient online measurements.

## 3 Methodology

**Fig. 1:**
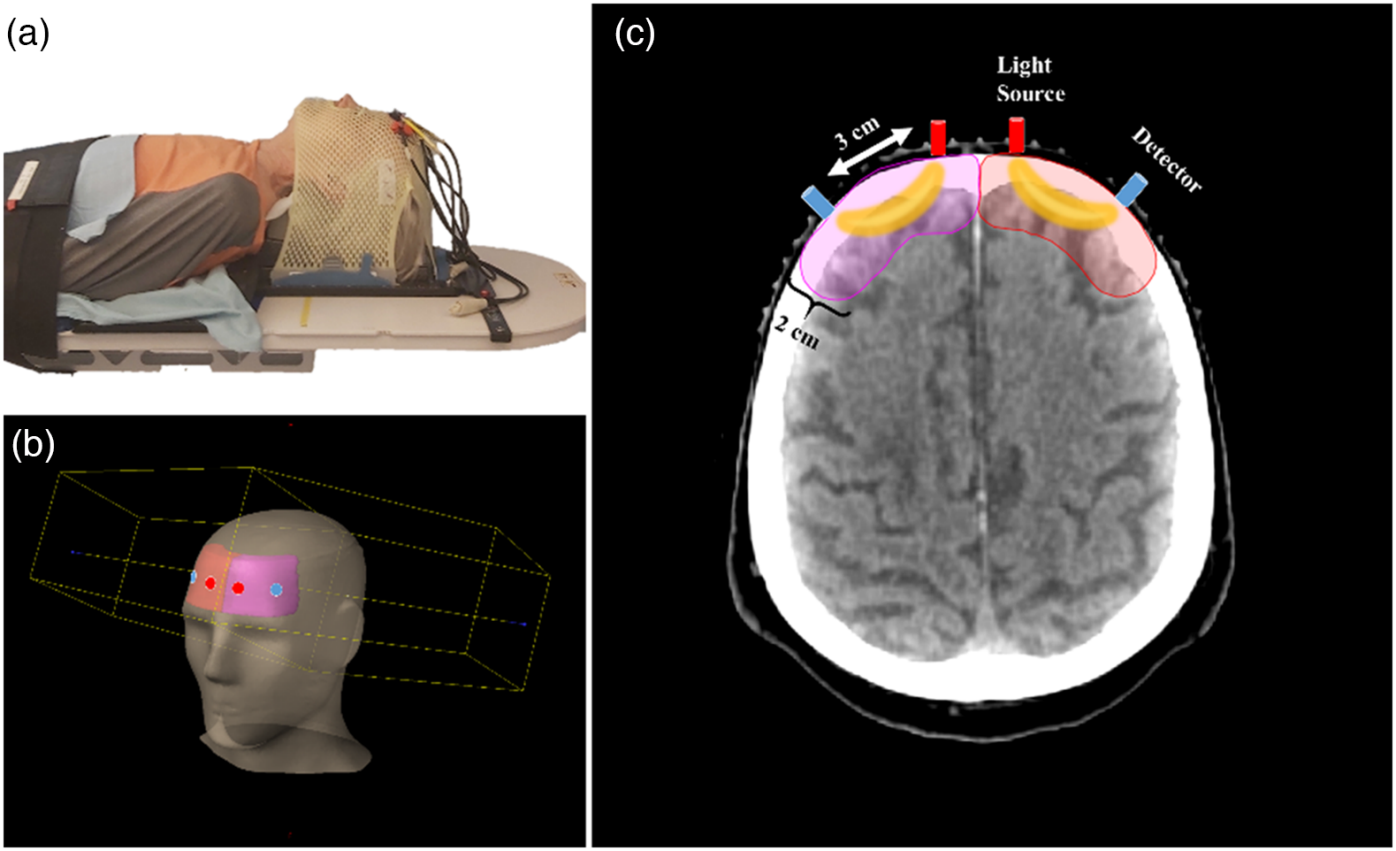
During the treatment, patients wear a plastic mesh mask to ensure the correct patient positioning to make the irradiation spatially accurate and to avoid patient movement. The mask is molded to perfectly fit the patient’s head shape. Multiple fibers can be easily attached to the mask for the fNIRS measurements. The fiber output tip (diameter of 2.5 mm) with a 90-deg bend are attached to forehead on left (NIRS ch1) and right (NIRS ch2) side using 3D printed fiber clips that are glued on the mask. (a) Patient is in the treatment position in the medical LINAC (TrueBeam, Varian Medical Systems), when optical fiber tips are attached perpendicular toward the brain at (b) source–detector distance of 3 cm and (c) the corresponding CT image.

The fNIRS device built for this study utilizes frequency coding technique, which is based on modulating the illuminating light, each wavelength at a specific frequency and demodulating correspondingly after receiving. Sampling rate of raw fNIRS signals was 1 kHz. Ionizing radiation inside the radiotherapy treatment room poses high demands on the materials and devices and because of this fNIRS device was placed outside radiotherapy room and only the optical fibers for the illumination and detectors we brought to the radiotherapy chamber from the control room by a cable entry. All optical fibers and optodes must be free of ferromagnetic metals since these will be attached on the subject’s head in the area of ionizing radiation. Fibers were 10 m long having two light source inputs and one output tip to enable mixing of the LED light sources each modulated at a specific frequency.^3^^,^^9^ This arrangement made it possible to ensure the radiotherapy compatibility of the fNIRS measurements. NIR light is produced by high-power LEDs manufactured by Roithner, at wavelengths of 690 and 830 nm. In the measurements (Fig. 1), we used two NIRS optodes attached on the subject’s forehead on both left (NIRS ch1) and right (NIRS ch2) side at the source–detector distances of 3 cm to ensure NIRS volume to reach the brain cortex.^10^

Measured raw NIRS time courses were converted into two-time courses representing temporal changes in HbO, HbR, and HbT concentrations using the modified Beer–Lambert law^11^ written in MATLAB. In the performed analysis, wavelengths of 690 and 830 nm were used. In addition, tissue oxygenation index (TOI) was calculated as a ratio between the HbO and HbT.^12^ TOI is an indicator of absolute tissue mixed with arterial and venous oxygen saturation. TOI is also named as the ${\mathrm{StO}}_{2}$ or regional oxygen saturation.

In the total 34 patients, radiotherapy measurements were analyzed from 10 patients (age 72.7±8.3  years, 4 females). All patients had a whole-brain radiotherapy treatment (WBRT) and radiation dose was delivered externally using LINACs. During the treatment session, the patient is in a supine position while radiation fields are targeted in the brain (see Fig. 1). All the measurements were performed with the LINAC (TrueBeam, Varian Medical Systems). Forward-intensity modulated radiation (FIMRT) technique was used with 6 MV x-ray beams at the dose rate of 600  MU/min. FIMRT is a dose delivery technique, which is used to provide more homogenous dose coverage to the whole brain area. As static field WBRT consists of two opposing radiation fields, the FIMRT adds smaller fields from the same direction of the main fields. This technique is also called field-in-field technique. In practice, in the case of WBRT, the opposing fields are on the sides of the patient. Due to the shape of the patient head and the goal to provide the desired dose in the center parts of the brain, dose maximum of the two opposing fields are generated on the frontal and on the occipital parts of the patient head. This is due to the oval shape of head so there is less mass in frontal and occipital parts of the head compared to the central parts. To avoid the dose maximums reaching levels outside the clinic dose coverage criteria (e.g., no maximum dose of 107% to 110%), smaller fields are generated by moving multileaf collimators to cover the frontal and/or occipital parts of the head and giving a smaller dose, compared to the main field, to the central area of the brain. Thus patient anatomy is the only reason to use the FIMRT technique to achieve the dose coverage criteria setup in clinic. In this study, FIMRT is not in the main role, in fact it complicates our measurement setup in comparison to static field irradiation. However, as most of the patients treated with WBRT are using FIMRT technique, exclusion of FIMRT patients would have drastically cut our sample size.

Our NIRS device can only measure the relative concentration instead of the absolute one. For this reason, different subjects will have different signal amplitudes. Since we need to combine signals from all subjects, we applied normalization by subtracting the signal value at the beginning of the radiation from the whole corresponding signals, i.e., HbO, HbR, HbT, and TOI. All processes were done after filtering the signal in the very low-frequency (VLF) band (0.008 to 0.1 Hz).

## 4 Results

**Fig. 2:**
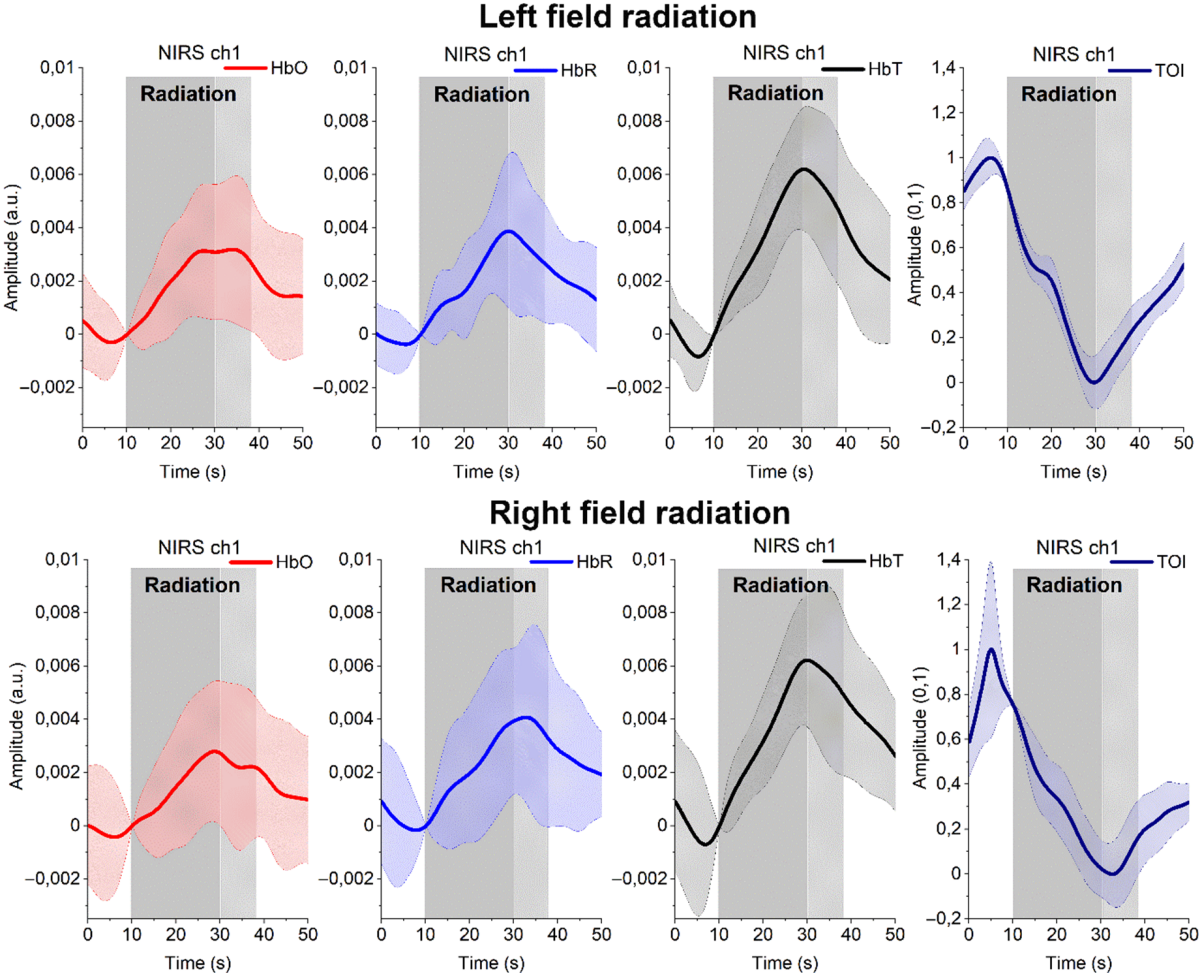
VLF band average (±std) responses of HbO (red), HbR (blue), and HbT (black) and TOI (on the right) to irradiation, colored with gray area. Dark gray (20 s) is the first irradiation that in this group analysis all patients had. The second irradiation, so-called FIMRT, and colored with light gray, was not given to all patients (see also Fig. 3). In all cases, due to radiation, HbT = HbO + HbR starts to increase reflecting cerebral blood volume increase, but at the same time, tissue oxygenation decreases, which can be seen as an almost linear change in the VLF band. (Note that TOI must be always 1 or less, however, its standard deviation can be higher, as is the case in these figures, because it measures the dispersion of a dataset relative to its mean calculated as the square root of the variance.)

**Fig. 3:**
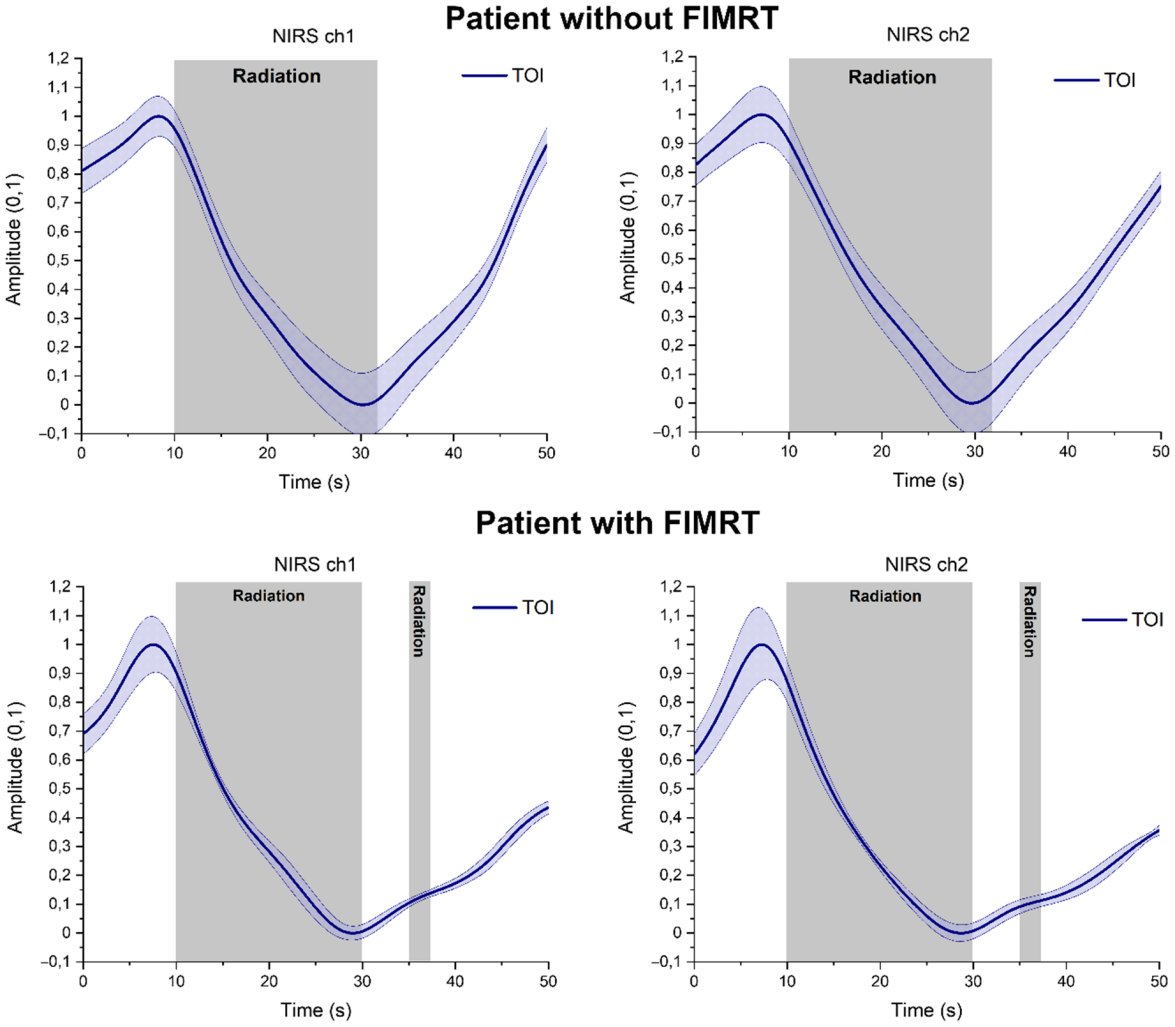
VLF band responses of TOI measured from forehead (left) and (right) in an individual patient with and without FIMRT. (Colored gray area shows when irradiation is on.) In particular, the response in patient with FIMRT is notable, because the second short-irradiation pulse causes small change in the TOI signal indicating that TOI parameter can accurately follow the irradiation induced tissue oxygenation level changes in real time. (Note that due to low-pass filtering effect of raw signals it looks like TOI starts to decrease before the irradiation starts. However, in raw signals, this change starts in synchrony along with the irradiation.)

In the following, we show in Fig. 2 average hemodynamic response including TOI to irradiation at VLF band, based on of 34 patient measurements. Next, Fig. 3 presents TOI at VLF band in two individual patients with and without FIMRT. In general, VLF band represents vasomotor response, which is associated with dilation/constriction of the vascular vessel, additionally, it removes effects of heartbeat and breathing. As can be seen in both figures, all signals show strong characteristic linear response to irradiation at VLF. As HbT reflects blood volume changes, it can be seen that, due to irradiation, blood volume increases thus suggesting increased blood flow. Although HbO increases correspondingly, TOI drops dramatically as a direct effect of irradiation and starts to increase again after the irradiation.

**Fig. 4:**
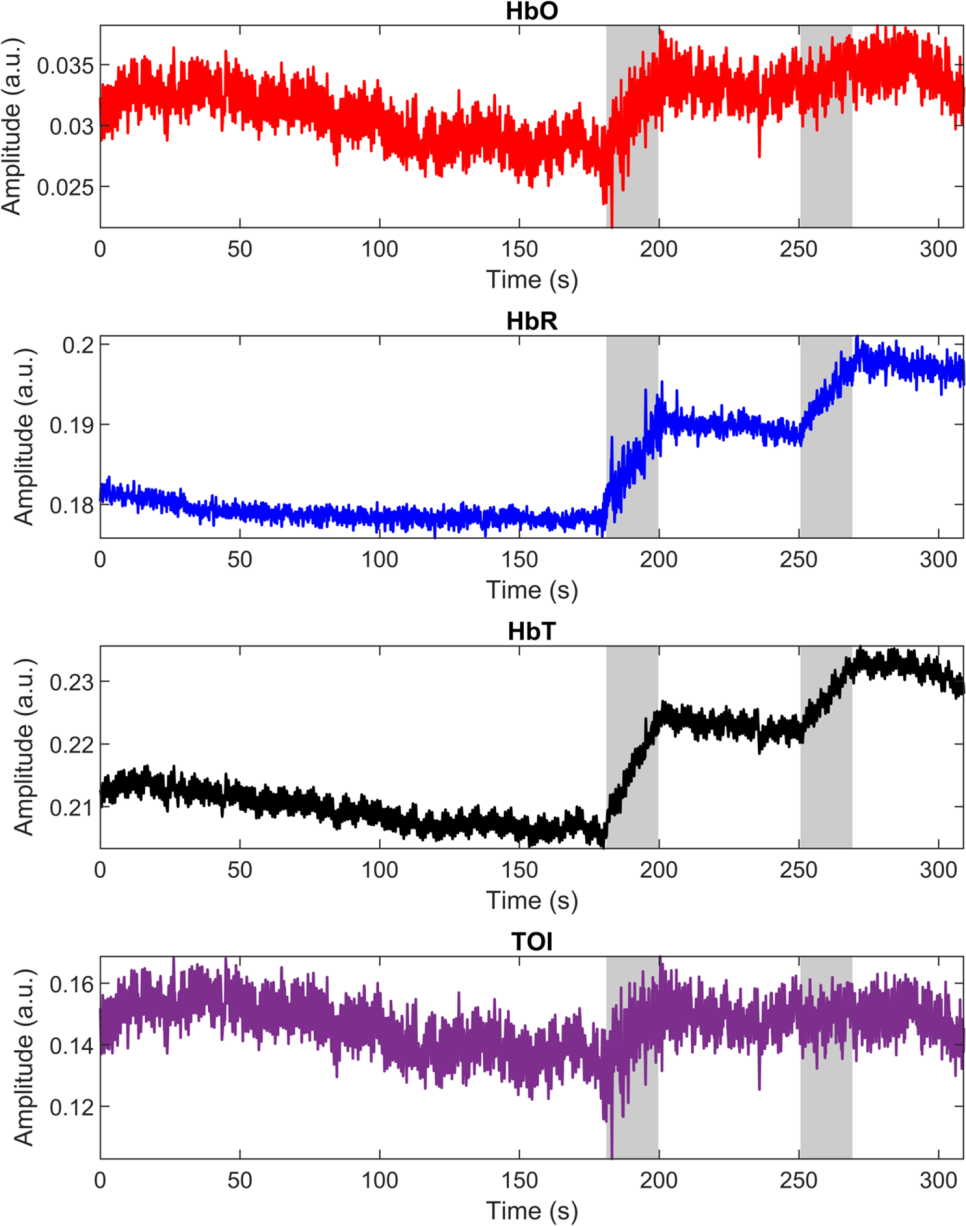
An example of a full band response measured from an individual patient during the whole radiotherapy treatment process. As can be seen, after the baseline measurement, hemodynamics are substantial when radiation treatment starts.

Figure 4 shows an example of a full band response measured from an individual patient during the whole 5 min radiotherapy treatment procedure. As can be seen, measured signals before and after starting the radiation are notable different.

## 5 Discussion

The WBRT or whole brain radiotherapy is palliative treatment, which is given to patients with identifiable brain metastases and prophylaxis for microscopic disease. In WBRT, the radiation dose is given in whole brain area only preventing dosage to eyes. We measured immediate cerebral haemodynamic and TOI changes caused by the WBRT. Radiation itself is not choosing the tissues it affects, the radiolysis process generates several end-products (especially reactive oxygen species), which cause reactions in chemical bonds of the tissue compounds, most notably DNA that itself can trigger responses in enzymes or immune systems. Consequently, we do not have physiological explanation of the response because there are no human or animal studies that we know of measuring these types of temporal real-time responses of irradiation. In addition, we cannot completely exclude involuntary physiological responses of patients to the external factors, as it would require “sham” treatments of the patients that our current ethical permission is not applicable. In this study, there were altogether 10 different patients, but the same subject went through several treatments that had the same treatment protocol. Altogether, 34 subject measurements were performed. However, it must be noted that these 34 measurements do not represent a set of independent observations. However, in order to ensure the creativeness of the measurement response, we conducted several verification fNIRS measurements by measuring radiation effects on water and blood samples inside vial. None of these caused changes in our fNIRS signals. Therefore, we can conclude that the presented fNIRS signal changes are visible only when measuring *in vivo* effect of radiation.

It seems the tendencies of tissue oxygenation are different for the first irradiation and the second irradiation. This effect could be due to multiple reasons. The main reason is being that the second irradiation is in much smaller scale in terms of absorbed dose compared to the first irradiation. Two irradiations in FIMRT technique with different absorbed doses in the WBRT are purely technical in order to provide more homogenous dose coverage in the whole brain. Furthermore, in the second irradiation, multileaf collimators move to the forehead (and sometimes back) of the patient giving dose only to the more central part of the brain, this means that the second irradiation is not only smaller dose but further away from our measuring range. Nevertheless, it seems that there could be some type of refractory period, which causes smaller or nonexistent response in back-to-back second irradiation. It remains to be elucidated whether this refractory period is due something physiological, originates from our measurement setup or is combination of both.

Previous studies monitoring tissue oxygenation, HbT and blood flow in the course of radiotherapy, suggest changes in blood flow and oxygen supply to the tumor in the first few weeks of the therapy.^1^^,^^7^^,^^13^ These longitudinal responses are in line with tumor and tissue responses mechanisms in radiotherapy, i.e., reoxygenation of the tumor cells. The tumor responses and tumor models indicate that the reoxygenation occurs due to tumor shrinkage, decreased oxygen consumption, and increased perfusion.^14^ However, our results showing instant responses in hemodynamics, particularly increased blood flow but strong decrease in TOI immediately due to the irradiation followed by disappearance of the effect after the radiation ends, suggest to the more immediate processes. This would have been unobservable in these previous studies as the measurements have been conducted between irradiations.

Hypoxia plays a crucial role in the tumor response to radiotherapy. The possibility to detect tissue oxygenation changes during the treatment process can lead to different strategies in radiation dose planning^15^ and potentially improve the outcome of radiation treatment.^16^

Our results display instant cerebral haemodynamic response recorded by fNIRS during clinical radiotherapy. Furthermore, this study shows that TOI is an accurate parameter to observe in real time the immediate tissue oxygenation effects also in individual patient [see Fig. 3 for the second irradiation (FIMRT)]. Based on the group analysis, fNIRS may offer an effective technique for continuous monitoring of tumor hypoxia in radiotherapy, which involves modulating tumor oxygenation to increase or decrease tumor hypoxia. This warrants still further fNIRS data collection and research in radiotherapy, because in our study, all patients had the same WBRT procedure and dose rate of 600  MU/min. Therefore, we cannot yet confirm if the fNIRS response correlates also with the level of dose rate, which will be our follow up study. There are small variations in responses between individual patients although the calculated irradiation dose is the same. In fact, we think this makes the presented monitoring technique especially interesting when aiming to tailor radiotherapy dose planning for individuals. Therefore, our next goal is to study more precisely the individual responses when using different dose rates.

## 6 Conclusion

In this study, we show that fNIRS measurement of cerebral hemodynamics in clinical radiotherapy is feasible to perform in regular basis. The irradiation induces immediate temporal alterations in cerebral hemodynamics, particularly a strong decrease in cerebral TOI. Potentially, presented monitoring technique can be used in future for improving dose planning and safety control for individual patients.
